# Supplementary material for: Assessment of Antioxidant and Antimicrobial Properties of Selected Greek Propolis Samples (North East Aegean Region Islands)
Source: Molecules. 2022 Nov 24;27(23):8198. doi: 10.3390/molecules27238198 (PMC9735805; doi:10.3390/molecules27238198)
Supplement: Supplementary file 1 [file molecules-27-08198-s001.zip › molecules-2043043-supplementary.pdf]

Assessment of Antioxidant and Antimicrobial Properties of Selected Greek Propolis Samples (North East Aegean Region Islands)

Elisavet Pyrgioti, Konstantia Graikou, Antigoni Cheilari and Ioanna Chinou *

Lab of Pharmacognosy and Chemistry of Natural Products, Faculty of Pharmacy, National and Kapodis-trian University of Athens, 15771 Zografou, Greece
* Correspondence: ichinou@pharm.uoa.gr

Table S1. GC-MS chemical analysis of the NEAR propolis extracts

**Substance %**	**PLs1**	**PLs2**	**PLs3**	**PLs4**	**PLs5**	**PLs6**	**PLs7**	**PLm1**	**PLm2**	**PCh1**	**PCh2**	**PFo**	**PPs**	**PIk**	**POi**	**PSa**
benzoic acid																0.21
butanedioic acid							0.50									
succinic acid	0.34			0.63				0.40	1.18			1.47				
methlymalonic acid								0.66								
hydrocinnamic acid	0.23			1.19					0.25			1.81				
decanoic acid										0.23		0.51				
malic acid								0.28				0.31				
sugar		0.37						2.59								
o-coumaric acid								0.48								0.37
2,3,4-thihydroxybutyric acid	0.30							0.75								
a-cedrol										0.51		1.39			0.61	
sugars	56.33	69.73	78.23	67.40	60.08	70.77	44.24	66.33	41.31	15.23	7.98	22.07	89.83	53.23		8.66
3-hydroxyvaleric acid									0.44							
p-hydrobenzoic acid	0.23							0.20				1.67		0.21		
sugar	3.30	2.59			3.57			3.40						0.40	38.56	
pentanedioic acid	0.59															
hexadecanoic acid							1.63				2.80					0.29
isobutyric acid		1.59														
sugar					4.97											

propanoic acid								1.96								
4-hydroxy-3-methoxybenzoic acid												0.21				
isovanillic acid														0.33		
azelaic acid										0.31		0.50				
2-hydroxyisovaleric acid					2.47											
lauric acid			0.81									0.24				
ethyl ester of oleic acid											0.79					
palmitic acid methyl ester										0.32						
sugar			0.95													
m-coumaric acid									4.76			0.22				
p-coumaric acid									4.07					0.27		
manoyl-oxide															0.25	
sugar	10.86							1.83	1.00	0.97					0.31	
3,4,5-trihydoxybenzoic acid	0.25							0.63								
cinnamic acid								0.45				0.31		3.31		1.39
sugar	1.01							5.82	1.48							
3,4-dimethoxycinnamic acid									0.80							0.32
gluconic acid	0.44							1.03								
palmitic acid	2.16	0.81	5.65	5.95	1.35	3.12		0.36	0.70	8.64		5.06	2.77	1.61	3.01	
manool														0.58		
sugar								0.42								
ferulic acid	0.38	0.24				0.54	0.97	0.50	2.22							
benzyl cinnamate														1.32		0.41
oleic acid methyl ester										0.33		0.28				
isoferulic acid									0.17							
caffeic acid	1.41	0.13		0.60		0.58	1.62	1.67	6.01							0.52
ethyl oleate	0.17		0.69										0.32			
communal															0.28	
octadecadienoic acid															0.72	

caffeic acid ester						0.36										
pentenyl-p-coumarate									0.82							
linoleic acid	0.42	0.24	0.90	0.84		0.47		0.16		1.43		0.75				0.3
oleic acid	2.20	1.19	6.14	4.27	1.13	2.34	2.40	0.63	0.87	6.07	3.12	5.79	3.34	1.55	0.89	0.56
stearic acid	0.23	0.13		0.38		0.29				0.80		0.47	0.17		0.54	
3-methyl-2-butenyl- ferulate																1.73
ferruginol									0.37						0.64	0.27
sugar									1.52							
diterpene (structure similar to totarol)															1.01	
2-hydroxyisocaproic acid	0.43															
diterpene (structure similar to isoagatholal)															2.88	
caffeic acid ester						0.57										
sempervirol										0.52		0.42		1.97		
14,15-dinor-13-oxo-8(17)-labden-19-oic acid												0.60		0.33	0.40	
diterpene												3.47				0.42
copalol										0.67				0.37	1.49	0.49
pimaric acid	0.49	0.61	0.68	0.54		0.63	0.85	0.71	0.46	1.19	1.88	2.87	0.26	0.45	0.76	
diterpene (structure similar to isoagatholal)														2.30		
diterpene (structure similar to pimaric acid)															6.72	
communic acid																1.79
totarol							1.55	0.43	0.35	4.03		3.94	0.18	0.16	0.54	4.89
pentenyl ferulate													0.19			
diterpene (structure similar to sempervirol)										2.53					0.54	
isopimaric acid	3.35	4.19	3.07	2.96	1.94		1.58	0.56		1.01	10.90	7.96		2.18		0.50

neoabietic acid		2.42				3.55				7.65	2.28				1.58	
diterpenic acid	1.10															
pimaric acid + imbricataloic acid	0.78					0.55				11.75		10.16		7.79	10.45	
13-epitorulosol																1.73
dehydroabietic acid	3.40	3.14	1.01	2.73	2.07	3.40		0.20		1.56	18.27	1.18	0.26	0.86	0.24	1.23
isopentyl caffeate									3.19							
diterpene		0.34												2.42		
3-methyl-2-butenyl caffeate	0.64		0.43	0.38		0.98	1.38	0.55	3.66							1.29
2-methyl-2-butenyl caffeate	0.33		0.45	1.18		0.38	1.56		0.75							0.44
3-methyl-3-butenyl caffeate	0.85	0.52		0.95		1.30		0.47	0.38							3.17
cinnamyl cinnamate														1.41		2.02
abietic acid	2.57	2.98		1.28	1.16	3.52		0.14		1.38	11.35	0.97		0.95		
13 epicupressic acid							4.45			0.66		1.87				8.07
totarolon										0.34		0.47		0.20		
isoagatholal							0.73			1.99		0.88		1.19	3.59	2.16
pinocembrin		0.21							0.40							
2-hydroxyferruginol											5.55	0.86				
hydroquinone											1.18					
dimethyl ether of kaempferol											10.60					
triterpene											2.14					
communal								0.19								
feruginolon																0.42
pinocembrin chalcone							2.18		0.43							0.85
agathadiol + imbricatoloic acid						0.27	1.88	0.35	0.15	4.32		2.84			5.50	2.88
15-hydroxy-dehydroabietic acid	0.27					0.47										
diterpene (structure similar to acetylisocupressic acid)															0.32	
agathadiol														1.62		2.70
isocupressic acid						0.84	8.60	0.19		15.62		8.58		8.26	7.39	14.08

diterpene (structure similar to isocupressic acid)															4.34	
diterpene															2.21	
13(14)-dehydrojunicedric acid									0.61							
sugar								0.69								
diterpenic acid								0.52								
pinobanksin	0.48						5.54		0.28							
junicedric acid						0.57				0.60		0.29			0.50	
acetylisocupressic acid										0.53					0.23	
sugar + ester of caffeic acid							3.60								0.18	1.52
dihydroxy-monomethoxy flavonone																0.68
alpinon chalcone																0.20
chrysin				0.65					0.78							0.63
flavanone																1.10
pinobanksin 3-o-acetate									0.41							0.59
1-o-octadecylglycerol	0.14		0.42				2.16			0.51	2.27	1.03	0.32			
galangin							1.13									0.60
pinostrobin chalcone									0.58							0.53
phenylethyl ester of caffeic acid							0.96		0.29							
dodecyl glycerol							0.49				1.11					
cinnamyl ester of caffeic acid									0.22							
benzyl coumarate																1.31
benzyl ester of caffeic acid																3.59
diterpene																0.62
chalcone																1.45

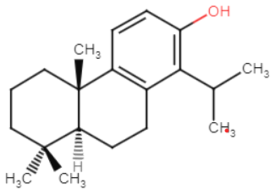

totarol

manoyl-oxide

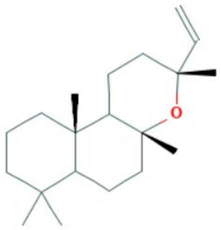

ferruginol

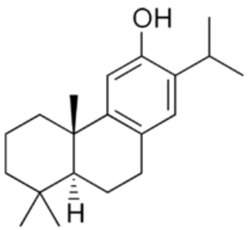

epitorulosol

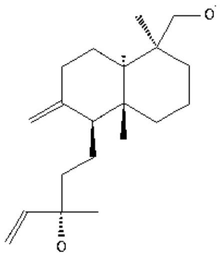

13-epitorreferol

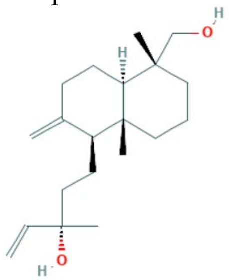

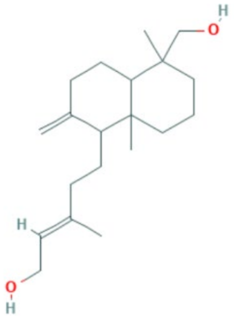

agathadiol

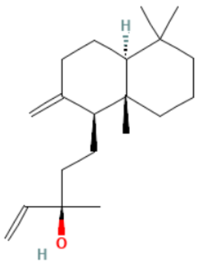

manool

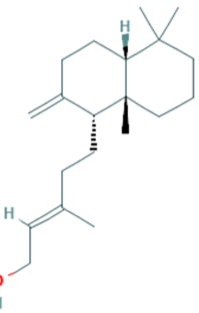

copalol

14,15-dinor-13-oxo-8(17)labden-19-oic acid

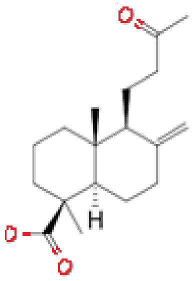

pimaric acid

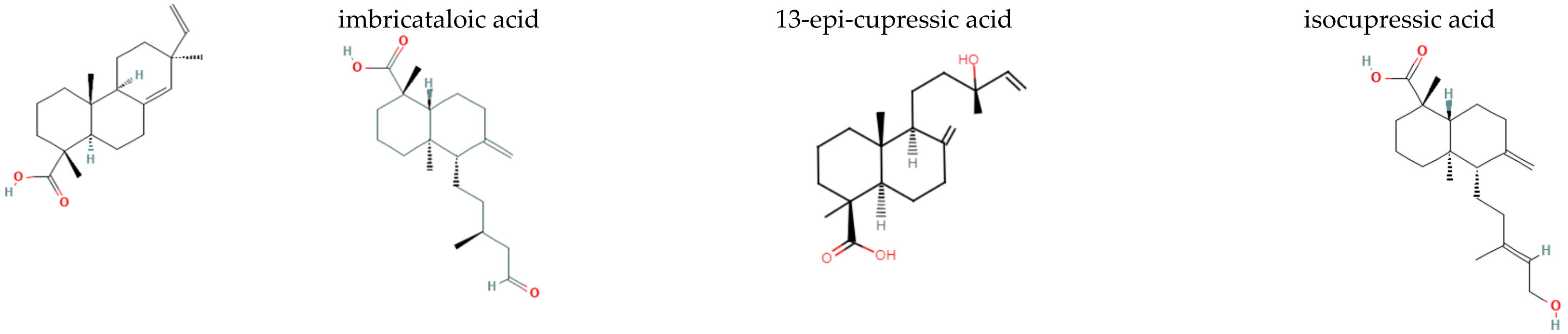

Figure S1. The chemical structures of the isolated compounds from NEAR propolis
